# A prediction model of dementia conversion for mild cognitive impairment by combining plasma pTau181 and structural imaging features

**DOI:** 10.1111/cns.70051

**Published:** 2024-09-18

**Authors:** Tao‐Ran Li, Bai‐Le Li, Jin Zhong, Xin‐Ran Xu, Tai‐Shan Wang, Feng‐Qi Liu, for the Alzheimer's Disease Neuroimaging Initiative

**Affiliations:** ^1^ Department of Neurology The First Affiliated Hospital of Nanjing Medical University, Jiangsu Province Hospital Nanjing China; ^2^ Department of Hematology The First Affiliated Hospital of Nanjing Medical University, Jiangsu Province Hospital Nanjing China; ^3^ Beijing Children's Hospital, Beijing Pediatric Research Institute Capital Medical University, National Center for Children's Health Beijing China; ^4^ Department of Neurology Yangzhou Friendship Hospital Yangzhou China

**Keywords:** Alzheimer's disease, biomarker, MCI, MRI, prediction model, pTau181

## Abstract

**Aims:**

The early stages of Alzheimer's disease (AD) are no longer insurmountable. Therefore, identifying at‐risk individuals is of great importance for precise treatment. We developed a model to predict cognitive deterioration in patients with mild cognitive impairment (MCI).

**Methods:**

Based on the Alzheimer's Disease Neuroimaging Initiative (ADNI) database, we constructed models in a derivation cohort of 761 participants with MCI (138 of whom developed dementia at the 36th month) and verified them in a validation cohort of 353 cognitively normal controls (54 developed MCI and 19 developed dementia at the 36th month). In addition, 1303 participants with available AD cerebrospinal fluid core biomarkers were selected to clarify the ability of the model to predict AD core features. We assessed 32 parameters as candidate predictors, including clinical information, blood biomarkers, and structural imaging features, and used multivariable logistic regression analysis to develop our prediction model.

**Results:**

Six independent variables of MCI deterioration were identified: apolipoprotein E ε4 allele status, lower Mini‐Mental State Examination scores, higher levels of plasma pTau181, smaller volumes of the left hippocampus and right amygdala, and a thinner right inferior temporal cortex. We established an easy‐to‐use risk heat map and risk score based on these risk factors. The area under the curve (AUC) for both internal and external validations was close to 0.850. Furthermore, the AUC was above 0.800 in identifying participants with high brain amyloid‐β loads. Calibration plots demonstrated good agreement between the predicted probability and actual observations in the internal and external validations.

**Conclusion:**

We developed and validated an accurate prediction model for dementia conversion in patients with MCI. Simultaneously, the model predicts AD‐specific pathological changes. We hope that this model will contribute to more precise clinical treatment and better healthcare resource allocation.

## Graphical abstract

Based on predictors that are routinely available, we provided an accurate and convenient diagnostic tool that can predict the clinical progression of mild cognitive impairment patients and identify Alzheimer's disease specific pathological changes. This tool may contribute to more precise clinical treatment and better health care resource allocation.

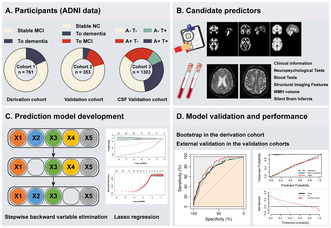

## 1 INTRODUCTION

Alzheimer's disease (AD) is a progressive neurodegenerative disease characterized by cognitive decline that affects millions of people worldwide and places an enormous burden on the public.^1^ The view that AD is irreversible is gradually being challenged. Recently, a series of encouraging clinical trial results have suggested the effectiveness of monoclonal antibodies, such as lecanemab and donanemab, in preventing disease progression in the early stages of AD.^2^, ^3^ Mild cognitive impairment (MCI) is the earliest recognizable stage of objective cognitive impairment in clinical practice and serves as an early warning of future dementia.^4^ However, most MCI cases are stable, and the annual conversion rate to dementia may be less than 10%.^4^, ^5^ Therefore, identifying the risk factors for cognitive decline and establishing a predictive model has substantial practical implications, especially since they would offer targets for interventions to delay or even halt disease progression.

In previous studies, we and other researchers used various complex methods to identify the early stages of AD or predict cognitive deterioration, such as extracting high‐dimensional features from multiparametric magnetic resonance imaging (MRI),^6^, ^7^ exploring brain glucose metabolic patterns using positron emission tomography (PET),^8^, ^9^ or analyzing amyloid‐β (Aβ) levels in neuronal‐derived extracellular vesicles.^10^ Although these models exhibit excellent discriminative abilities, their practical applications are limited. The pathological core features of AD, including the extracellular deposition of Aβ and the intraneuronal presence of aggregated hyperphosphorylated tau proteins,^11^ as well as various non‐specific features of neuroinflammation and synaptic dysfunction, can all be quantified using cerebrospinal fluid (CSF) or PET analysis, and their levels are significantly correlated with cognitive ability.^12^ However, these methods are expensive and invasive, making them more suitable for confirmation than prediction. Alternatively, blood biomarkers and structural MRI are more clinically feasible.

In recent years, blood biomarkers have become increasingly popular with the development of platforms for hypersensitive detection.^13^, ^14^ For example, increased neurofilament light (NFL) levels in blood appear to reflect the severity of AD‐related neurodegeneration,^15^ and blood phosphorylated tau contributes to the recognition of AD‐specific pathologies across the cognitive continuum.^16^ These biomarkers have the potential to serve as valuable prognostic and susceptibility markers. The anatomical features on MRI are classic markers of neurodegeneration. In addition, a close connection between AD and cerebrovascular injuries has been emphasized.^17^ Similar to macroscopic infarcts, cerebral microangiopathy contributes substantially to dementia.^18^ Therefore, imaging features of cerebral small vessel disease may also indicate future cognitive decline.^19^

The combination of blood and MRI biomarkers holds significant potential for improving the accuracy of predicting dementia conversion, given their complementary nature in capturing different aspects of the disease. In this study, based on a sample of well‐characterized participants, we aimed to: (1) propose a novel, practical, and convenient multivariable model for predicting cognitive deterioration based on basic clinical, blood, and imaging features while further establishing a quantitative risk scoring system; and (2) evaluate the model's ability to estimate AD core features. Previous predictive models either lacked practical application value or exhibited poor performance.^7^, ^20^, ^21^, ^22^ We hope to provide an accurate and convenient diagnostic tool that will ultimately lead to more appropriate referrals and interventions for patients with early‐stage AD.

## 2 MATERIALS AND METHODS

### 2.1 Participants and study design

Data used in the preparation of this article were obtained from the Alzheimer's Disease Neuroimaging Initiative (ADNI) database (adni.loni.usc.edu). The ADNI was launched in 2003 as a public‐private partnership, led by Principal Investigator Michael W. Weiner, MD. The primary goal of ADNI has been to test whether serial MRI, PET, other biological markers, and clinical and neuropsychological assessment can be combined to measure the progression of MCI and early AD. For up‐to‐date information, see http://www.adni‐info.org.

**FIGURE 1:**
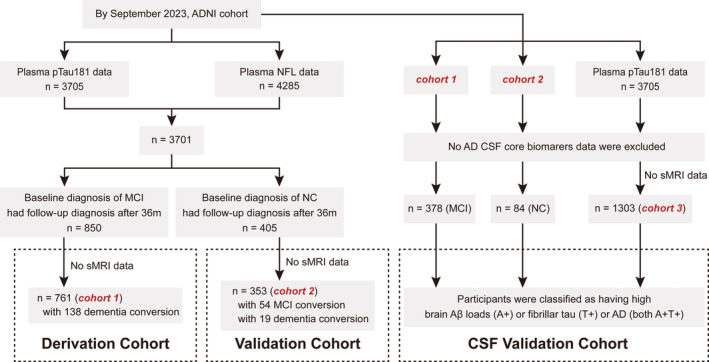
Formation of the cohorts. We aimed to establish a clinical model for predicting future cognitive deterioration based on clinical information, blood biomarkers, and sMRI indicators. By September 2023, 761 participants were selected, called Cohort 1. This cohort was used to screen candidate predictor variables and build prediction models (derivation cohort). Cohort 2 included 353 NCs for model validation (validation cohort). Furthermore, participants without CSF data were excluded to form the CSF validation cohorts, including a subset of Cohort 1, a subset of Cohort 2, and a re‐screened cohort of 1303 participants (Cohort 3). They were classified as having high brain Aβ loads (A+) or fibrillar tau (T+) according to a priori principles; AD+ means both A+ and T+. AD, Alzheimer's disease; ADNI, Alzheimer's Disease Neuroimaging Initiative; Aβ, amyloid‐β; CSF, cerebrospinal fluid; m, month; MCI, mild cognitive impairment; NCs, cognitively normal controls; NFL, neurofilament light; pTau181, phosphorylated‐tau181; sMRI, structural magnetic resonance imaging.

Figure 1 illustrates the screening process of the participants. Briefly, we used Cohort 1 as a derivation cohort to select candidate predictor variables and construct prediction models and Cohort 2 as an independent validation cohort. The participants in the derivation cohort were diagnosed with MCI at baseline. They had available plasma NFL, tau phosphorylated at residue 181 (pTau181), and structural MRI data and underwent clinical follow‐up after 36 months. By contrast, the participants in the validation cohort were cognitively healthy at baseline. In addition, we screened participants with available AD CSF core biomarkers to form the “CSF validation cohorts” to clarify the model's ability to predict AD core features (A/T profile), which are in accordance with the latest research framework.^13^, ^23^ All enrolled participants provided detailed clinical information, including demographic data, apolipoprotein E (*APOE*) status, and neuropsychological test results. The tests included the Clinical Dementia Rating (CDR) scale, Mini‐Mental State Examination (MMSE), Logical Memory test, etc. The ADNI database classified individuals clinically as cognitively normal controls (NCs; MMSE score: ≥24, CDR score: 0), those with MCI (MMSE score: ≥24, CDR score: 0.5, and objective memory loss measured using education‐adjusted scores on delayed recall of logical memory), or those with AD dementia following predefined criteria.^24^

### 2.2 CSF biomarkers

Alzheimer's disease core biomarkers, including CSF Aβ_42_, pTau181, and total tau levels, were measured using fully automated Roche Elecsys® immunoassays as described previously.^25^ Participants in the CSF validation cohorts were classified as having high brain Aβ loads (A+) or fibrillar tau (T+) according to predefined principles that utilized established cutoff values of <977 pg/mL for CSF Aβ_42_ and >27 pg/mL for phosphorylated tau.^26^ Participants were considered to have core AD features (AD+) if they were both A+ and T+. Details regarding CSF collection and detection are available at http://adni.loni.usc.edu/.

### 2.3 Plasma biomarkers

The measurement procedures for plasma pTau181 and NFL levels have been comprehensively described in previous publications.^27^, ^28^ Briefly, the assay was based on a single‐molecule array method using a combination of commercial monoclonal antibodies. The concentrations used in this study were all above the lower limit of detection.

### 2.4 Neuroimaging acquisition and processing

Structural imaging was performed using a 1.5‐ or 3.0‐T MRI scanner with a 3D T1‐weighted MPRAGE sequence. A region‐of‐interest analysis using FreeSurfer (version 4.3 for 1.5‐T data and version 5.1 for 3.0‐T data; http://surfer.nmr.mgh.harvard.edu/) was performed to obtain regional morphological parameters, including volume and cortical thickness. The results were included only if they passed quality control. Previous studies have emphasized structural indicators related to cognitive deterioration and AD.^29^, ^30^, ^31^, ^32^ Based on this, we considered the volume of the hippocampus and amygdala and the thickness of the entorhinal cortex, fusiform gyrus, inferior parietal lobule, inferior temporal gyrus, middle temporal gyrus, and parahippocampal gyrus as potential predictor variables.

White matter hyperintensities (WMH) and silent brain infarcts are the classical imaging hallmarks of cerebral small vessel disease.^33^ These were considered candidate predictor variables in the current study. The measurements were performed in the DeCarli Lab (UC‐Davis). For WMH, a Bayesian approach was used to segment the high‐resolution 3D T1 and fluid‐attenuated inversion recovery (FLAIR) sequences.^34^ Briefly, images were processed to (1) exclude non‐brain tissues, (2) spatially align, and (3) remove MRI field artifacts. The images were then warped to a standard template space where the prior probability of WMH occurrence and the FLAIR signal characteristics of the WMH were modeled at every location in the cerebral white matter. This prior information, together with the signal intensities of the FLAIR images in question, was used to identify WMH. With the help of an image analysis system,^35^ which allows simultaneous projection of the complete imaging sequence dataset at three times magnified, infarcts were identified by experienced neurologists using T1‐weighted and FLAIR MRI images (≥3 mm). According to previous classification methods,^36^, ^37^, ^38^ the participants were grouped based on the presence or absence of infarcts, the number of infarcts (none, single, or multiple), and the presence or absence of infarcts at a specific anatomical site, such as the thalamus, cortex, or infratentorial structure.

Details regarding imaging acquisition protocols and processing are available online (http://adni.loni.usc.edu).

### 2.5 Candidate predictors and study outcome

We aimed to establish a convenient model for predicting future cognitive deterioration with practical implications. Candidate predictor variables for inclusion in our models were selected based on a literature review, and we also considered the feasibility of utilizing the ADNI database.^11^, ^12^, ^15^, ^16^, ^19^, ^29^, ^30^, ^31^, ^32^ Before model development, all predictor variables were evaluated for correlations to avoid multicollinearity in the multivariable logistic analysis.

The outcome of the study was the deterioration in cognitive function after 36 months. Currently, AD is recognized as a distinct entity and is defined pathologically by the presence of specific A/T profiles^13^, ^23^; thus, we also analyzed the model's ability to predict the core features of AD.

### 2.6 Statistical analysis

Basic participant characteristics were summarized as numbers (%) or means (standard deviations) for categorical and continuous variables, respectively. Groups were compared using chi‐square tests for categorical variables and independent‐sample *t*‐tests for continuous variables.

The sample size for this study was based on feasibility considerations, and a post hoc sample calculation was performed. According to the guidelines for fitting multivariable models, we used the following formula: *N* = (*n* × 15)/*I*, where *N* represents the sample size, *n* represents the number of variables in the final multivariable logistic model (*n* = 6), and *I* represents the incidence of conversion from MCI to dementia within 36 months. Considering the reported conversion rates of 6.0% to 44.8% in the literature^4^, ^5^ and the 18.1% rate observed in our data, we set the rate at 20.0%. Thus, the test results demonstrate that the sample size in our study was sufficient to develop a prediction model.

#### 2.6.1 Prediction model development

We evaluated 32 independent candidate variables for their potential to predict future cognitive deterioration. We performed univariable and multivariable logistic regression analyses to assess the effects of these candidate predictors. Variables associated with MCI deterioration (*p* < 0.1) in the univariable analysis were included in the multivariable regression analysis. Two independent and parallel methods were used to develop a parsimonious predictor model while minimizing overfitting: stepwise backward variable elimination and least absolute shrinkage and selection operator (Lasso) regression, with a significance level of 0.05 for variable retention. The overall goodness‐of‐fit of the models was assessed using the Akaike Information Criteria (AIC) and likelihood ratio tests. Model discrimination was compared based on *C*‐statistics, integrated discrimination improvement (IDI), and net reclassification index (NRI). A risk heatmap and a nomogram were established based on multivariable logistic regression analysis. Moreover, we integrated variables predictive of dementia conversion into a clinical score using the multivariable logistic regression model. We assigned a risk point value for each variable proportional to the respective β coefficients. All β coefficients were standardized so that the lowest one had a point value of 0.5 to make the risk scores close to an integer and facilitate intuitive use.

#### 2.6.2 Prediction model validation

We internally validated the prediction model in the derivation cohort using the bootstrap method with 1000 repetitions and further externally validated the prediction model in the validation and CSF validation cohorts.

#### 2.6.3 Prediction model performance

We assessed discrimination, calibration, and net benefit to evaluate the performance of the model. Discrimination was calculated using concordance statistics (area under the receiver operating characteristic curve [AUC]). Calibration was assessed using the Hosmer–Lemeshow test and visualized with the calibration plot. A perfect plot is indicated by the 45° diagonal line. To further investigate the model's clinical utility, decision curve analysis (DCA) was performed to determine the net clinical benefit.

All statistical analyses were performed using the R programming language (version 4.2.1) and GraphPad Prism (version 9.4.0).

## 3 RESULTS

### 3.1 Participant characteristics

A total of 761 participants with MCI were included in the derivation cohort (Cohort 1; Figure 1). Of them, 432 (56.8%) were men, and the average age was 72.9 years (range: 55.2–92.6 years). At the 36th month, 138 (18.1%) participants developed dementia. As a subset, 575 individuals (75.6%) were evaluated for cerebral WMH and infarcts, and there were no differences between the two groups (Table S1). Three hundred and fifty‐three NCs, with a similar proportion of men (49.0%), were included in the validation cohort (Cohort 2; Figure 1). The average age was 76.3 years (range: 56.1–95.3 years). At the 36th month of follow‐up, 73 participants (20.7%) had cognitive deterioration, of whom 54 developed MCI and 19 developed dementia. Detailed demographic and clinical characteristics are presented in Table 1.

**TABLE 1:** Baseline characteristics of the derivation and validation cohorts.

	Derivation (cohort 1)	Validation (cohort 2)
	(*n* = 761)	(*n* = 353)
Demographics		
Age, mean (SD), years	72.86 (7.673)	76.33 (6.755)
Men	432 (56.8%)	173 (49.0%)
Education, mean (SD), years	16.15 (2.669)	16.27 (2.572)
*APOE* ε4 allele		
ε4 −/−	438 (57.6%)	254 (72.0%)
ε4 +/−	253 (33.2%)	96 (27.2%)
ε4 +/+	70 (9.2%)	3 (0.8%)
MMSE, mean (SD), score	28.06 (1.834)	29.01 (1.304)
Plasma biomarkers		
NFL, mean (SD), pg/mL	38.92 (23.710)	39.67 (18.897)
pTau181, mean (SD), pg/mL	17.31 (10.895)	17.03 (15.086)
Structural MRI		
V_L_Hippocampus, mean (SD), %^a^	0.231 (0.0432)	0.242 (0.0343)
V_R_Hippocampus, mean (SD), %^a^	0.237 (0.0435)	0.243 (0.0355)
V_L_Amygdala, mean (SD), %^a^	0.089 (0.0187)	0.090 (0.0152)
V_R_Amygdala, mean (SD), %^a^	0.093 (0.0185)	0.095 (0.0162)
T_L_Inferior Temporal Cortex, mean (SD), mm	2.709 (0.2022)	2.704 (0.1923)
T_R_Inferior Temporal Cortex, mean (SD), mm	2.747 (0.1966)	2.747 (0.1926)
Diagnosis at baseline	MCI	NC
Conversion after 36 months	Dementia: 138 (18.1%)	MCI: 54 (15.3%) Dementia: 19 (5.4%)

Abbreviations: APOE, apolipoprotein E; L, left; MCI, mild cognitive impairment; MMSE, mini‐mental state examination; MRI, magnetic resonance imaging; NC, cognitively normal control; NFL, neurofilament light; pTau181, phosphorylated‐tau181; R, right; SD, standard deviation; T, thickness; TIV, total intracranial volume; V, volume.

^a^ Volume of the hippocampus and amygdala was presented as the ratio of the regional volume to TIV, multiplied by a factor of 100.

Patients with available AD CSF core biomarkers were selected as the CSF validation cohorts (Figure 1), which included subsets of Cohorts 1 (*n* = 378), 2 (*n* = 84), and 3 (*n* = 1303). Cohort 3 contained these two subsets and consisted of 457 NCs, 635 patients with MCI, and 211 patients with dementia. Table S2 presents the detailed information. The proportions of A+, T+, and AD+ participants in the subset of Cohort 1 were 50.8%, 34.1%, and 26.2%, respectively; those in the subset of Cohort 2 were 35.7%, 28.6%, and 16.7%, respectively; and those in Cohort 3 were 52.1%, 38.7%, and 29.2%, respectively.

### 3.2 Identifying independent risk factors for conversion from MCI to dementia

In the derivation cohort, univariable analysis identified a significant correlation between MCI deterioration and 22 risk factors consistent with previous reports (Table S3): older age, lower years of education, *APOE* ε4 allele status, lower scores on the MMSE scale, higher levels of plasma NFL and pTau181, and all selected MRI indicators (bilateral). Each significant variable was included in a multivariable logistic regression model using a backward elimination procedure or a Lasso regression (Figure S1). After screening, the Lasso method resulted in one fewer variable, the thickness of the right inferior temporal cortex, compared with observations in the previous method. Age was excluded by both methods. Table 2 lists the performances of the models. The six‐variable model (Model 1) derived from the backward method was found to be the most appropriate. Adding an additional predictor age (Model 2) did not improve the model, with no significant changes in fit (likelihood ratio test: *p* = 0.052; AIC: 541.6 vs. 539.8), *C*‐index (0.848 vs. 0.851, *p* = 0.346), IDI (*p* = 0.219), and NRI (*p* = 0.597). By contrast, deleting the predictor thickness of the right inferior temporal cortex (Model 3) worsened the model, with significant changes in fit (likelihood ratio test: *p* < 0.001; AIC: 541.6 vs. 557.5), *C*‐index (0.848 vs. 0.832, *p* = 0.015), IDI (−2.59%, *p* = 0.003), and NRI (approaching significance, *p* = 0.160).

**TABLE 2:** Performance of models in the derivation cohort.

	Model 1: *APOE* ε4 status, MMSE, V_L_Hippocampus, V_R_Amygdala, T_R_Inferior Temporal Cortex, Plasma pTau181 levels	Model 2: Model 1 plus Age	Model 3: Model 2 delete T_R_Inferior Temporal Cortex
Source of model	Stepwise backward	Stepwise backward (plus age)	Lasso (plus age)
Akaike Information Criterion	541.6	539.83	557.5
Comparison	Model 2 vs. model 1	Model 3 vs. model 2	Model 3 vs. model 1
Fitting degree			
*p* Value (likelihood ratio)	0.052	< 0.001	< 0.001
*C* statistic	0.848 (0.815–0.882)	0.851 (0.820–0.884)	0.832 (0.802–0.870)
*p* Value (bootstrap)	0.346	0.003	0.015
IDI (%)	−0.450 (−1.160 to 0.260)	−3.040 (−4.740 to −1.330)	−2.590 (−4.280 to −0.910)
*p* Value	0.219	< 0.001	0.003
NRI	−0.018 (−0.081 to 0.050)	−0.096 (−0.188 to 0.021)	−0.078 (−0.178 to 0.036)
*p* Value (bootstrap)	0.597	0.077	0.160

*Note*: A bootstrap method of 1000 iterations were used to compare the *C*‐index and NRI between models.

Abbreviations: APOE, apolipoprotein E; IDI, integrated discrimination improvement; L, left; Lasso, least absolute shrinkage and selection operator; MMSE, mini‐mental state examination; NRI, net reclassification index; pTau181, phosphorylated‐tau181; R, right; T, thickness; V, volume.

Finally, six independent variables associated with MCI deterioration were identified by multivariable logistic regression analysis: *APOE* ε4 allele status, lower MMSE scale scores, higher plasma pTau181 levels, smaller volume of the left hippocampus and right amygdala, and reduced thickness of the right inferior temporal cortex (Table 3).

**TABLE 3:** Final multivariable logistic regression model of dementia conversion for patients with MCI in the derivation cohort.

	*β*	SE	*p* Value	OR (95% CI)
Constant	16.172			
*APOE* ε4 allele				
ε4 −/−				1 (reference)
ε4 +/−	0.890	0.247	<0.001	2.436 (1.503–3.971)
ε4 +/+	1.559	0.350	<0.001	4.756 (2.389–9.471)
MMSE score	−0.294	0.059	<0.001	0.746 (0.663–0.836)
V_L_Hippocampus (%)^a^	−10.009	3.548	0.005	0.000045 (0.000–0.046)
V_R_Amygdala (%)^a^	−20.732	8.609	0.016	0.000001 (0.000–0.018)
T_R_Inferior Temporal Cortex (mm)	−2.453	0.620	<0.001	0.086 (0.025–0.285)
Plasma pTau181 levels (pg/mL)	0.028	0.010	0.006	1.029 (1.008–1.050)

Abbreviations: APOE, apolipoprotein E; L, left; MCI, mild cognitive impairment; MMSE, mini‐mental state examination; pTau181, phosphorylated‐tau181; R, right; T, thickness; TIV, total intracranial volume; V, volume.

^a^ Volume of the hippocampus and amygdala was presented as the ratio of the regional volume to TIV, multiplied by a factor of 100.

### 3.3 Developing a prediction model

The equation for the prediction model for conversion from MCI to dementia is as follows:

$$\text{Probability}\;\left(\text{dementia}\right)=\frac{\exp\;\left(Y\right)}{1+\exp\;\left(Y\right)}$$

where:

$$\begin{array}{c} \begin{array}{c} Y=16.172\;+\;0.890\;\times \;\text{APOE}\;\varepsilon 4\;\text{allele count of}\;1\\ \;+\;1.559\;\times \;\text{APOE}\;\varepsilon 4\;\text{allele count of}\;2\;-\;0.294\\ \;\times \;\text{MMSE score}\;-\;10.009\\ \;\times \;\text{Intracranial volume ratio of left hippocampus}\;\left(\%\right)\;-\;20.732\\ \;\times \;\text{Intracranial volume ratio of right amygdala}\;\left(\%\right)\;-\;2.453\\ \;\times \;\text{Cortical thickness of right inferior temporal gyrus}\;+\;0.028\\ \;\times \;\text{Plasma pTau}181\;\text{levels} \end{array} \end{array}$$

**FIGURE 2:**
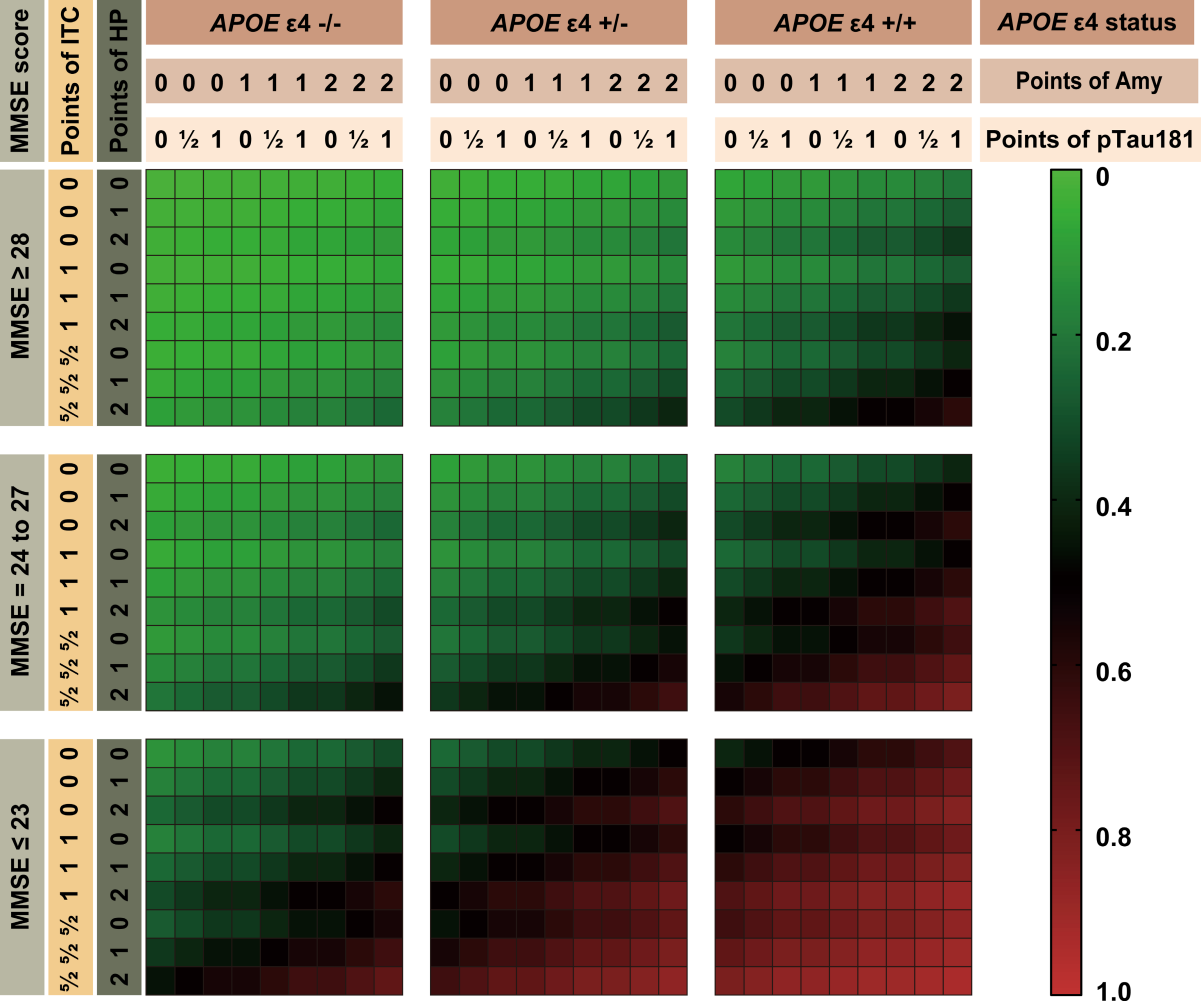
The risk estimation of dementia conversion in patients with MCI. Chart based on six included parameters: *APOE* ε4 status, MMSE score, intracranial volume ratio of left hippocampus (%), intracranial volume ratio of right amygdala (%), cortical thickness of right inferior temporal cortex, and plasma pTau181 levels. APOE, apolipoprotein E; MCI, mild cognitive impairment; MMSE, mini‐mental state examination; pTau181, phosphorylated‐tau181.

We developed a risk score system based on multivariable logistic regression. We assigned point values to each parameter based on the *β* coefficient: an *APOE* ε4 allele count of zero scored 0 points, one scored 2 points, and two scored 4 points; an MMSE score ≥28 scored 0 points, a score between 24 and 27 scored 2.5 points, and a score ≤23 scored 5.5 points; an intracranial volume ratio of the left hippocampus ≥0.250% scored 0 points, between 0.201% and 0.249% scored 1 point, and ≤0.200% scored 2 points; an intracranial volume ratio of the right amygdala ≥0.100% scored 0 points, between 0.081% and 0.099% scored 1 point, and ≤0.080% scored 2 points; cortical thickness of the right inferior temporal cortex (mm) ≥2.800 scored 0 points, between 2.601 and 2.799 scored 1 point, and ≤2.600 scored 2.5 points; plasma pTau181 levels (pg/mL) ≤12,000 scored 0 points, between 12,001 and 19,999 scored 0.5 points, and ≥20,000 scored 1 point (Table 4). The prediction estimates of the model and risk score distributions are shown in Figure S2 and Table S4. The lowest score (0 points) and the highest score (17.0 points) indicated a 1.5% and 93.3% risk of MCI deterioration, respectively. Moreover, the risk heatmap and nomogram of the model are provided for convenient use in clinical practice (Figure 2; Figure S3).

**TABLE 4:** The model score for prediction of MCI deterioration.

Risk factor	Points
*APOE* ε4 allele	
ε4 −/−	0
ε4 +/−	2
ε4 +/+	4
MMSE score	
≥28	0
24–27	2.5
≤23	5.5
Intracranial volume ratio of left hippocampus (%)	
≥0.250	0
0.201–0.249	1
≤0.200	2
Intracranial volume ratio of right amygdala (%)	
≥0.100	0
0.081–0.099	1
≤0.080	2
Cortical thickness of right inferior temporal cortex (mm)	
≥2.800	0
2.601–2.799	1
≤2.600	2.5
Plasma pTau181 levels (pg/mL)	
≤12,000	0
12,001–19,999	0.5
≥20,000	1

Abbreviations: APOE, apolipoprotein E; MCI, mild cognitive impairment; MMSE, mini‐mental state examination; pTau181, phosphorylated‐tau181.

### 3.4 Evaluating the performance of the model

**FIGURE 3:**
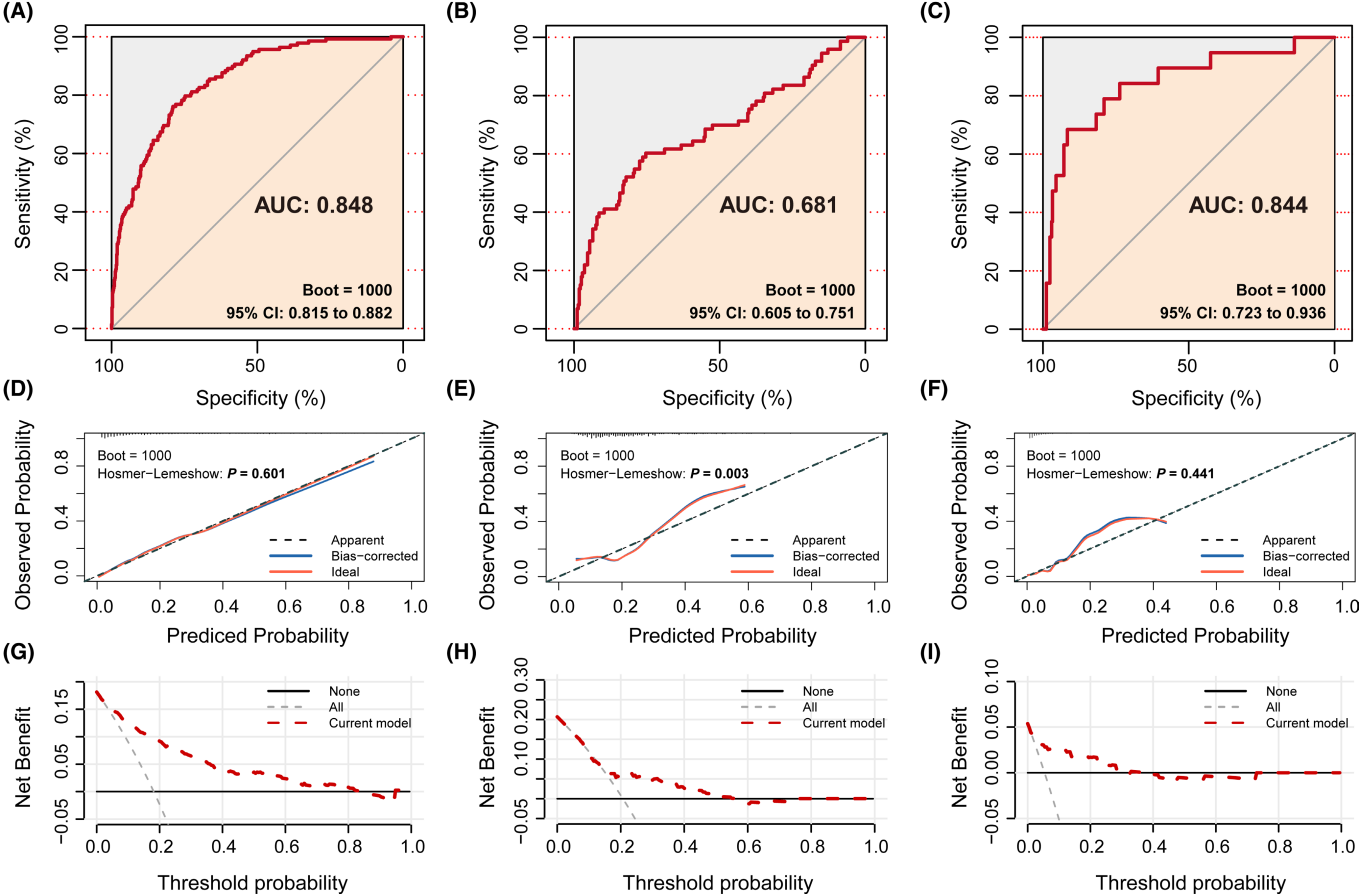
The predictive accuracy, calibration plot and decision curve analysis of the model for predicting clinical deterioration. The predictive accuracy of the model for predicting clinical deterioration (A–C). (A) ROC curve of the clinical model for predicting dementia conversion for patients with MCI in the derivation cohort (cohort 1); the AUC is 0.848 (95% CI 0.815–0.882). (B) ROC curve of the model for predicting cognitive deterioration (MCI or dementia) for NCs in the validation cohort (cohort 2); the AUC is 0.681 (95% CI 0.605–0.751). (C) ROC curve of the model for predicting dementia conversion for NCs in the validation cohort (cohort 2); the AUC is 0.844 (95% CI 0.723–0.936). Calibration plot of the clinical model for predicting cognitive deterioration (D–F). (D) model in the derivation cohort (cohort 1). (E) Model in the validation cohort (cohort 2; MCI or dementia conversion for NCs). (F) Model in the validation cohort (cohort 2; dementia conversion for NCs). The model‐predicted probability of cognitive deterioration was plotted on the *x*‐axis; actual probability was plotted on the *y*‐axis. An ideal calibration plot is indicated by a 45° diagonal line; Hosmer‐lemeshow test value (*p*) was listed. Decision curve analysis of the clinical model for predicting clinical deterioration in (G), the derivation cohort (cohort 1), (H) validation cohort (cohort 2; MCI or dementia conversion for NCs), and (I) validation cohort (cohort 2; dementia conversion for NCs). Black line: assumes no patient has clinical deterioration. Gray dashed line: assumes all patients have clinical deterioration. These two lines serve as a reference. AUC, area under the curve; CI, confidence interval; MCI, mild cognitive impairment; NC, cognitively normal control; ROC, receiver operating characteristic.

To evaluate the performance of the model, we first conducted an internal validation using bootstrap resampling with 1000 repetitions (Figure 3A,D). The model exhibited strong discrimination in predicting conversion from MCI to dementia, with an AUC of 0.848 (95% CI 0.815–0.882). The calibration plots demonstrated excellent agreement between the predicted probability and the actual observations. We further validated the model in an independent cohort (Cohort 2). The AUC of the model for predicting conversion from NCs to MCI or dementia was 0.681 (95% CI 0.605–0.751), demonstrating moderate discrimination capability (Figure 3B). The calibration curves also deviated from the diagonal and did not achieve statistical significance (*p* < 0.05; Figure 3E). However, if we focus only on dementia conversion, the performance is excellent, with an AUC of 0.844 (95% CI 0.723–0.936; Figure 3C). Figure 3F shows the calibration curve, which is statistically significant (*p* > 0.05), indicating good consistency between the predicted and actual observations. Finally, we assessed the net benefit of the intervention strategy using the risk prediction model compared to a treat‐all or treat‐none strategy. The DCA indicated a positive net benefit in the internal validation, starting from a 10% probability threshold (Figure 3G). However, the analysis suggested that the benefit window in the external validation was relatively small (Figure 3H,I), likely due to the different baseline diagnoses.

**FIGURE 4:**
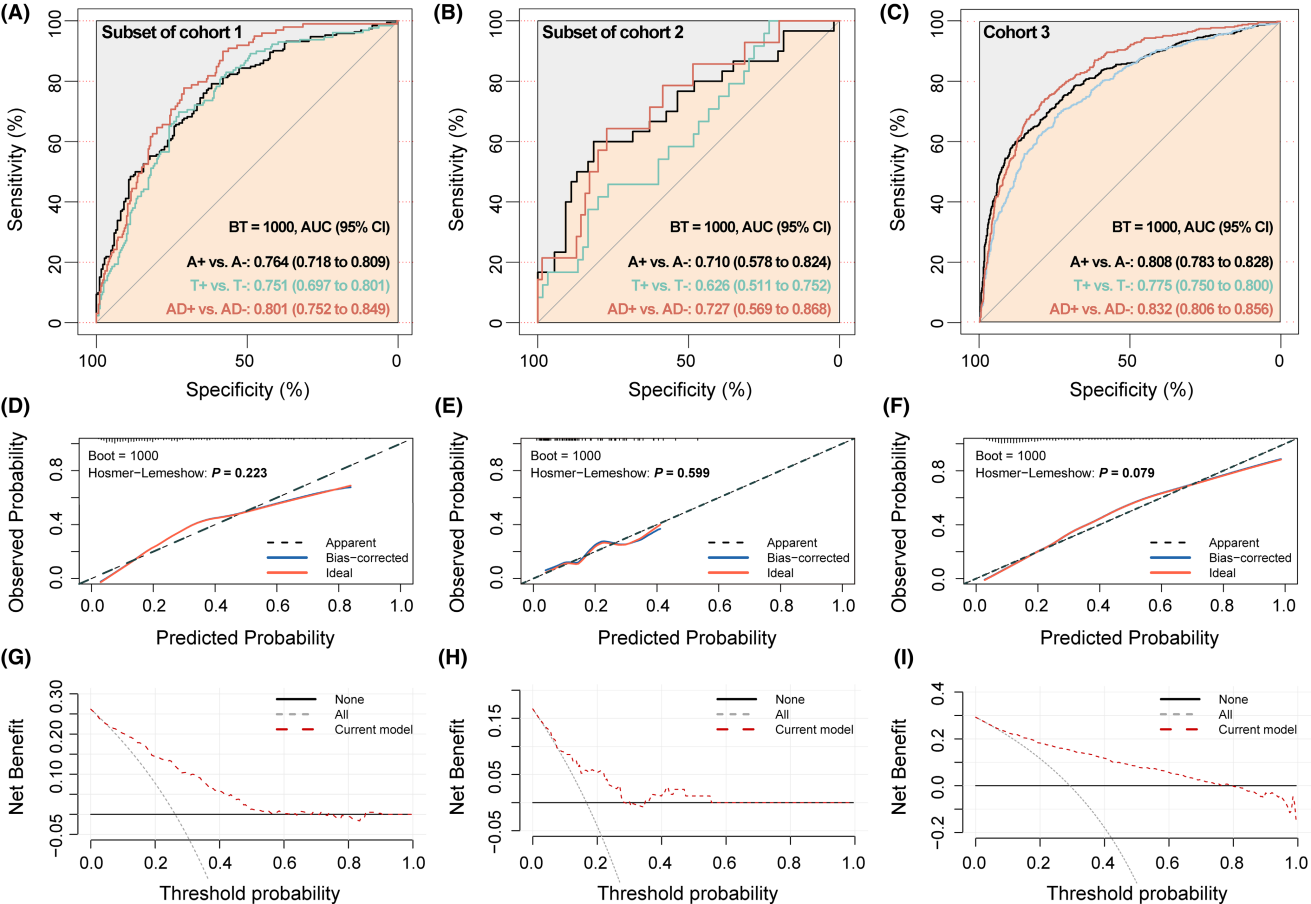
The predictive accuracy, calibration plot and decision curve analysis of the model for predicting AD CSF core features. The predictive accuracy of the model for predicting AD CSF core features (A+, T+, and AD+) in CSF validation cohorts (A–C). (A) Subset of cohort 1, (B) subset of cohort 2, (c) Cohort 3. Calibration plot of the clinical model for predicting AD+ subjects (D–F). (D) model in the subset of cohort 1, (E) model in the subset of cohort 2, (F) model in the cohort 3. The model‐predicted probability of cognitive deterioration was plotted on the *x*‐axis; actual probability was plotted on the *y*‐axis. An ideal calibration plot is indicated by a 45° diagonal line; Hosmer‐lemeshow test value (*p*) was listed. Decision curve analysis of the clinical model for predicting AD+ subjects in (G), subset of cohort 1, (H) subset of cohort 2, and (I) cohort 3. Black line: assumes no patient has clinical deterioration. Gray dashed line: assumes all patients have clinical deterioration. These two lines serve as a reference. Participants were classified as having high brain Aβ loads (A+) or fibrillar tau (T+) according to a priori principles; AD+ means both A+ and T+. AD, Alzheimer's disease; AUC, area under the curve; CI, confidence interval; CSF, cerebrospinal fluid.

The performance of the model in predicting AD core features is shown in Figure 4A–C. Specifically, its accuracy in discriminating A+ participants from A− participants reached 0.764 (95% CI 0.718–0.809), 0.710 (95% CI 0.578–0.824), and 0.808 (95% CI 0.783–0.828) in the subset of Cohort 1, Cohort 2, and Cohort 3, respectively. The ability to recognize T+ participants was slightly lower, with AUCs ranging from 0.626 to 0.775. For further recognition of AD+ participants, the AUC was 0.801 (95% CI 0.752–0.849), 0.727 (95% CI 0.569–0.868), and 0.832 (95% CI 0.806–0.856), respectively. Figure 4D–F indicates that the AD+ prediction accuracy was close to the actual values (all *p* > 0.05). DCA indicated a broad positive net benefit, especially in the subsets of Cohorts 1 (Figure 4G) and 3 (Figure 4I), but a narrow benefit window in the subset of Cohort 2 (Figure 4H). Furthermore, participants in Cohort 3 were divided according to diagnosis, and the results showed that the model's discriminatory capability was acceptable (Table S5), particularly in the MCI subset (A+ vs. A−: AUC 0.784 [95% CI 0.751–0.816]; T+ vs. T−: AUC 0.765 [95% CI 0.727–0.802]; AD+ vs. AD−: AUC 0.808 [95% CI 0.770–0.843]).

## 4 DISCUSSION

Generally, AD is irreversible once it reaches the dementia stage because the degree of neuronal loss becomes irreparable.^11^ By contrast, the early stages may be partially reversible with timely and appropriate treatment.^2^, ^3^ Identifying patients with unstable MCI is crucial for precise treatment and, ultimately, for reducing the social burden. In the present study, we established a prediction model based on clinical information, blood biomarkers, and structural MRI indicators. Internal validation using a bootstrap of 1000 iterations suggested that the model could reliably predict dementia conversion in patients with MCI at the 36‐month mark. External validation suggested that the model could also predict future cognitive deterioration in NCs and identify participants with AD‐specific pathologies.

For imaging features, we selected brain regions closely related to memory, such as the medial temporal lobe, as well as regions susceptible to AD‐specific pathological invasion, such as the entorhinal cortex and the neocortex, in accordance with previous studies.^29^, ^30^, ^31^, ^32^ After univariate logistic regression screening, 22 variables were retained, all of which were well‐established risk factors. Unexpectedly, both WMH and silent infarcts were eliminated. The connection between WMH, cognitive decline, and dementia has been well‐established in several large meta‐analyses.^39^, ^40^, ^41^ Cross‐sectional studies have suggested that WMH are associated with cognitive function across all major domains.^42^ According to several postmortem investigations, individuals with dementia and pure AD pathologies are uncommon; the majority of patients frequently exhibit mixed alterations involving both AD and vascular pathologies.^43^, ^44^ Each thrombotic event promotes Aβ production,^45^ and the prevailing view is that cerebrovascular lesions lower the threshold for AD symptoms.^17^, ^46^ However, it should be noted that participants in the ADNI dataset typically have a low vascular risk burden, resulting in small brain lesions. Additionally, we focused on the total volume of WMH rather than their patterns (punctuated or confluent) or locations (deep or periventricular), and cognitive decline may only be associated with specific patterns and locations of WMH.^47^, ^48^

In the multivariate analysis, we used stepwise backward variable elimination and Lasso regression to address multicollinearity and simplify variables. We then compared models in terms of fit and discrimination ability. The resulting model integrates genetic susceptibility, objective cognitive evaluation, structural indicators, and plasma biomarkers of pTau181, which complement one another, allowing the model to balance simplification with differentiation. Theoretically, blood NFL reflects the severity of atrophy, hypometabolism, and the decline in white matter integrity, particularly in regions typically affected by AD.^15^ Its levels begin to rise 6.8 to 15 years before the expected symptom onset in patients with familial AD.^49^, ^50^ However, this was not included in our prediction model. This omission is due to NFL being primarily a marker of neurodegeneration, possibly with less weight compared with that of MRI structural abnormalities. Age was likely excluded for similar reasons. The final model included the volumes of the hippocampus and amygdala and the thickness of the inferior temporal cortex. These results align with observations from clinical practice and several prior studies demonstrating early and profound atrophy in these regions among patients with MCI and dementia.^31^, ^51^ To avoid missing information, we included both left and right indicators rather than their summations. Previous studies on AD diagnosis and prediction also indicated a biased advantage.^6^, ^9^, ^30^, ^52^ The specific reason for this remains unclear and may be related to brain laterality.

Several prediction models have been developed for dementia conversion in patients with MCI. Zhao et al. added hippocampal radiomic features related to changes in MMSE scores sequentially into a logistic regression, improving the AUC of the basic prediction model from 0.65 to 0.82.^7^ A recently published study systematically reviewed the application of machine learning in predicting dementia conversion^20^ and found that the average accuracy was approximately 75%, while more complex models, such as those based on deep learning combined with multimodal and multidimensional data, achieved better performance, with accuracy rates exceeding 90%. However, for clinicians, these methods are obscure, and the features obtained are difficult to interpret. Moreover, deep learning is an end‐to‐end black box that lacks interpretable features.^53^ On the other hand, modeling using overly simple features decreases classification accuracy. Li et al. established prediction models based on demographic, medical history, lifestyle, and cognitive data, finding that the AUC for logistic regressions ranged from 0.64 to 0.70.^21^ Buratti et al. focused on indicators related to carotid atherosclerosis using ultrasonographic assessment, but their model achieved only approximately 70% accuracy, even in the derivation dataset.^22^ Our prediction model withstood internal validation and demonstrated reliable results in terms of AUC, calibration plot, and DCA. However, the ability and accuracy of the model in predicting cognitive deterioration in NCs were poor. By contrast, when only dementia conversion was considered, the classification results were excellent, with an AUC of 0.844. Despite this, DCA still showed a narrow benefit window. From our perspective, the use of NCs instead of individuals with MCI for external verification is suboptimal. The dementia conversion rate in NCs was significantly lower than in patients with MCI. Moreover, the selected predictors, especially the MMSE score, were likely unsuitable for NCs. Phenotype is a manifestation of intrinsic pathology. We found that this model was capable of recognizing the core CSF features of AD. The accuracy of predicting A+ and AD+ cases was over 80% among participants with different cognitive levels (Cohort 3). Similar efficacy was achieved in the MCI subgroup, indicating that the model identified both cognitive deterioration and pathological changes simultaneously.

This study has several limitations. First, according to the ADNI inclusion/exclusion criteria, the participants were mostly white, highly educated, and had relatively low vascular risk burdens. In the future, it will be necessary to verify the prediction model using other datasets. Second, this study was a retrospective analysis and thus bears the inherent limitations of such studies. Further prospective studies with larger cohorts are warranted. Third, due to the limitations of the dataset, we did not consider blood Aβ, an indicator of intracranial Aβ deposition,^13^, ^14^ or blood glial fibrillary acidic protein, an indicator of astrocytic activation,^13^ as candidate predictor variables. The inclusion of these markers may further improve the model. Fourth, the sample size of the NCs may have been insufficient because of the low dementia conversion rate. Validation requires a large sample of NCs. Fifth, although the model's ability to predict AD CSF core features is already prominent, this value is likely underestimated due to changes in outcome events. It is difficult for a single model to balance these two outcomes.

## 5 CONCLUSIONS

At the 2023 Alzheimer's Association International Conference, the value of blood markers was highlighted to an unprecedented extent and will be further popularized through market promotion.^13^ In this context, we aimed to establish a model for predicting cognitive deterioration using typical blood markers and conventional structural MRI. Our model includes predictors that are routinely available and easily accessible for incorporation into computer systems within healthcare services, or for integration into an application. We provided an easy‐to‐use and convenient nomogram and risk heatmap that can be easily adopted in clinical practice, in addition to a risk score. In summary, we developed and validated a highly discriminative, well‐calibrated, and parsimonious prediction model to predict dementia conversion risk in participants without dementia. The model also has the ability to predict AD pathological core features, allowing for early diagnosis and intervention, ultimately contributing to more precise clinical care and better healthcare resource allocation.

## AUTHOR CONTRIBUTIONS

**Tao‐Ran Li:** Supervision; resources; conceptualization; data curation; formal analysis; methodology; funding acquisition; writing – original draft; writing – review and editing. **Bai‐Le Li:** Data curation; methodology; writing – review and editing. **Jin Zhong** and **Xin‐Ran Xu:** Methodology. **Tai‐Shan Wang:** Writing – review and editing. **Feng‐Qi Liu:** Supervision; resources; conceptualization; methodology; project administration; funding acquisition; writing – review and editing. All authors reviewed the manuscript. The authors read and approved the final manuscript.

## FUNDING INFORMATION

This work was supported by grants “82300149” from the National Natural Science Foundation of China, grants “2023M741462” from the China Postdoctoral Science Foundation, grants “MXJL202211” from the First Affiliated Hospital of Nanjing Medical University, grants “PY2023018” from the Young Scholars Fostering Fund of the First Affiliated Hospital of Nanjing Medical University, and grants “2023ZB308” and “2023ZB182” from the Department of Human Resources and Social Security of Jiangsu Province.

## CONFLICT OF INTEREST STATEMENT

On behalf of all authors, the corresponding author confirms no conflict of interest. All authors agreed to the publication of the manuscript in its current form.

## Supporting information


Data S1.

## Data Availability

The datasets used and/or analyzed during the current study are available from the corresponding author on reasonable request.
