# Supplementary material for: A prediction model of dementia conversion for mild cognitive impairment by combining plasma pTau181 and structural imaging features
Source: CNS Neurosci Ther. 2024 Sep 18;30(9):e70051. doi: 10.1111/cns.70051 (PMC11410557; doi:10.1111/cns.70051)
Supplement: Supplementary file 1 — Data S1. [file CNS-30-e70051-s001.docx]

**Supplementary Information for**

***A Prediction Model of Dementia Conversion for Mild Cognitive Impairment by Combining Plasma pTau181 and Structural Imaging Features***

**Table of Contents**

| **Content** | **Page** |
| --- | --- |
| **Supplementary Figures** |  |
| **Figure S1**. LASSO regression model | **2** |
| **Figure S2**. Modeled risk score distribution (bars) and predicted dementia conversion (line) in the entire cohort | **2** |
| **Figure S3**. Nomogram to estimate the probability of dementia conversion | **3** |
| **Supplementary Tables** |  |
| **Table S1**. Baseline Characteristics of the Derivation Cohort | **4** |
| **Table S2**. Baseline Characteristics of the CSF Validation Cohorts | **5** |
| **Table S3**. Univariable analysis of predictors associated with dementia conversion for patients with MCI in the derivation cohort | **6** |
| **Table S4**. Predicted risk of dementia conversion according to the model score | **8** |
| **Table S5**. ROC curves | **9** |


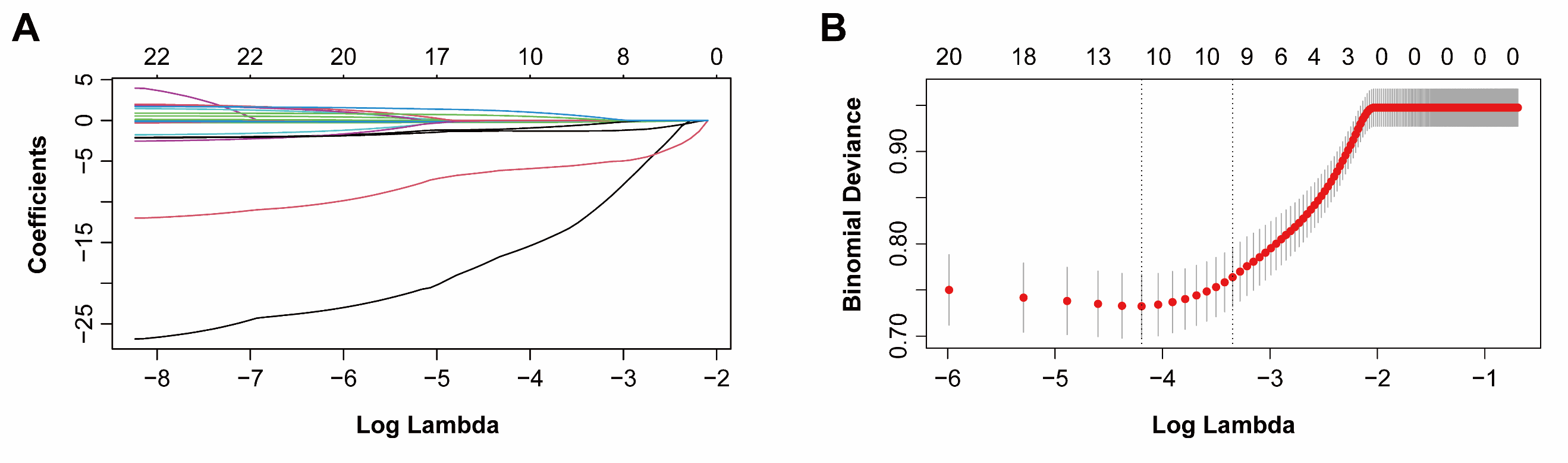


**Figure S1. LASSO regression model.**

**A**, LASSO coefficient profiles of the 22 candidate variables. Each curve represents a coefficient, and the x-axis represents the regularization penalty parameter. As λ changes, a coefficient that becomes non-zero enters the LASSO regression model. **B**, Five-fold cross-validation to select the optimal tuning parameter (λ). The left vertical line represents the minimum error, and the right vertical line represents the cross-validated error within 1 standard error of the minimum.

Abbreviations: LASSO, least absolute shrinkage and selection operator.


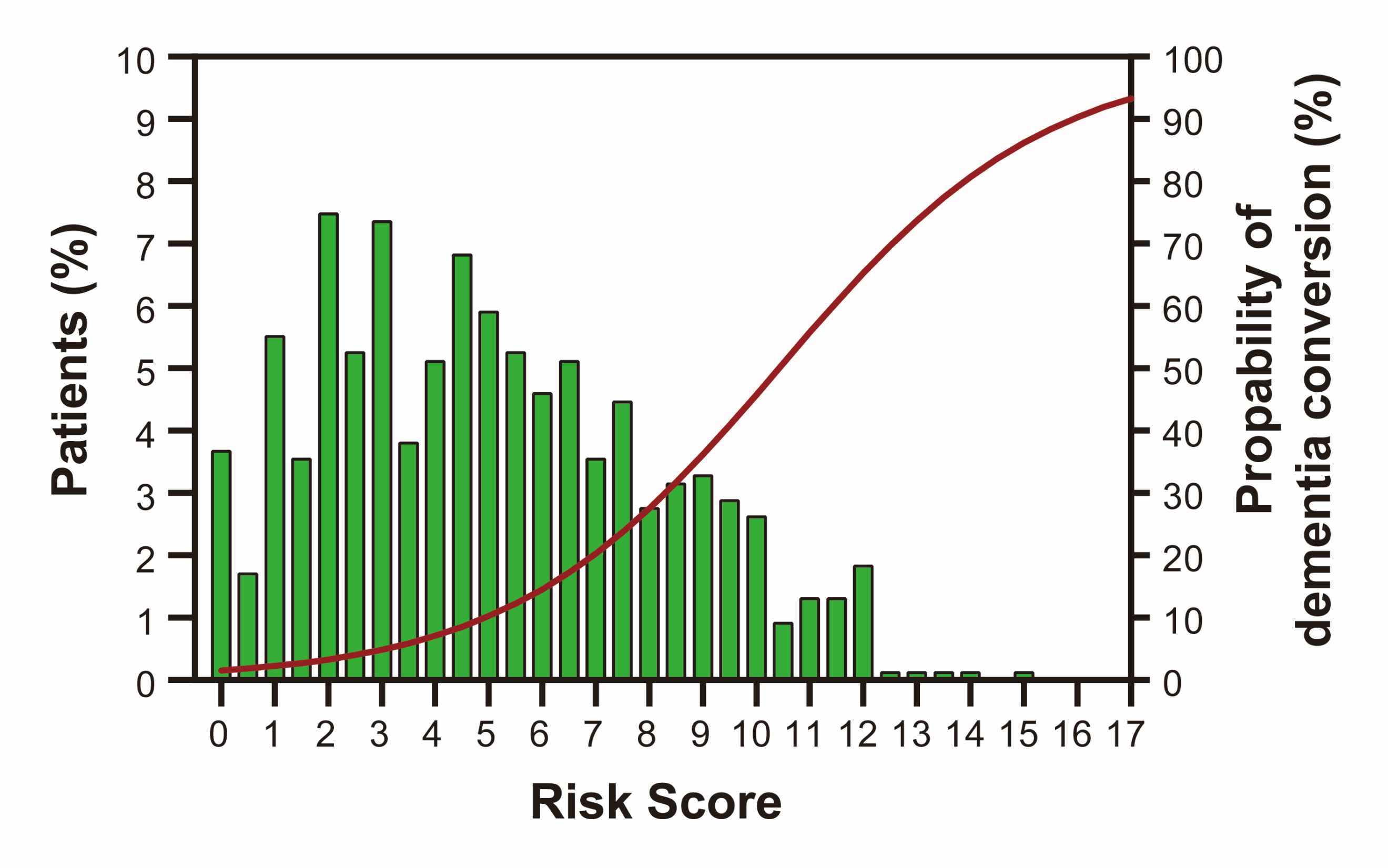


**Figure S2. Modeled risk score distribution (bars) and predicted dementia conversion (line) in the entire cohort.**


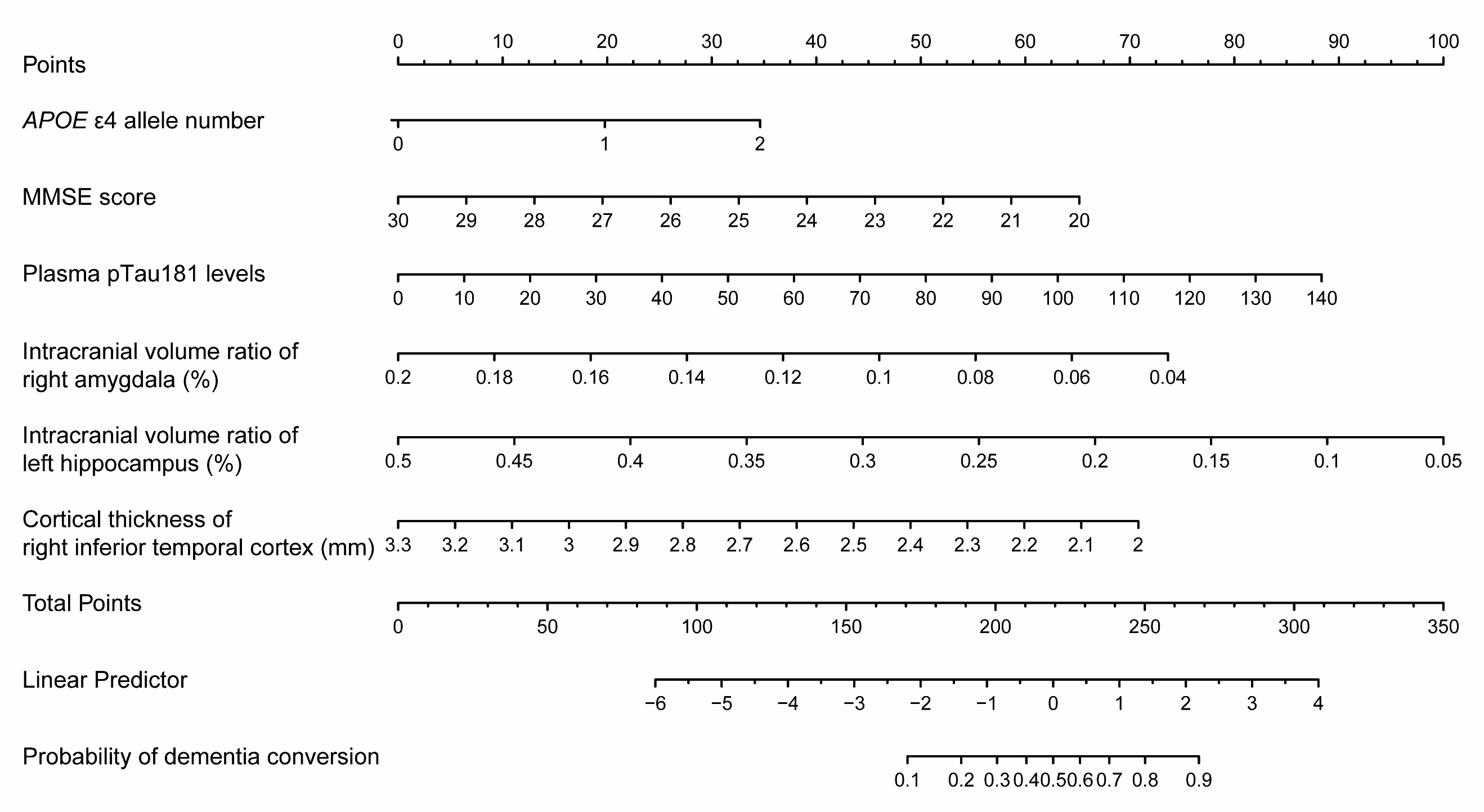


**Figure S3. Nomogram to estimate the probability of dementia conversion.**

To use the nomogram, an individual patient’s values are located on each variable axis, and a line is drawn upward to determine the number of points received for each variable value. Then, add the points from all of the variables and draw a line from the total points axis to determine the probabilities of dementia conversion at the lower line of the nomogram.

**Table S1. Baseline Characteristics of the Derivation Cohort**

|  | **Cohort 1**  **(n = 761)** | **Subset of Cohort 1**  **(n = 575)** |
| --- | --- | --- |
| **Demographics** |  |  |
| Age, mean (SD), y | 72.86 (7.673) | 72.13 (7.475) |
| Men | 432 (56.8%) | 324 (56.3%) |
| Education, mean (SD), y | 16.15 (2.669) | 16.28 (2.581) |
| ***APOE* ε4 allele** |  |  |
| ε4 -/- | 438 (57.6%) | 330 (57.4%) |
| ε4 +/- | 253 (33.2%) | 188 (32.7%) |
| ε4 +/+ | 70 (9.2%) | 57 (9.9%) |
| **MMSE, mean (SD), score** | 28.06 (1.834) | 28.05 (1.792) |
| **Plasma biomarkers** |  |  |
| NFL, mean (SD), pg/mL | 38.92 (23.710) | 37.37 (22.868) |
| pTau181, mean (SD), pg/mL | 17.31 (10.895) | 16.88 (10.061) |
| **Structural MRI** |  |  |
| V_L_Hippocampus, mean (SD), %^§^ | 0.231 (0.0432) | 0.232 (0.0438) |
| V_R_Hippocampus, mean (SD), %^§^ | 0.237 (0.0435) | 0.238 (0.0425) |
| V_L_Amygdala, mean (SD), %^§^ | 0.089 (0.0187) | 0.089 (0.0181) |
| V_R_Amygdala, mean (SD), %^§^ | 0.093 (0.0185) | 0.094 (0.0184) |
| T_L_Inferior Temporal Cortex, mean (SD), mm | 2.709 (0.2022) | 2.707 (0.2013) |
| T_R_Inferior Temporal Cortex, mean (SD), mm | 2.747 (0.1966) | 2.755 (0.1944) |
| **Diagnosis at baseline** | MCI | MCI |
| **Conversion after 36 months** | Dementia: 138 (18.1%) | Dementia: 102 (17.7%) |

The subset was additionally evaluated for cerebral WMH and infarcts. Mark §: volume of the hippocampus and amygdala was presented as the ratio of the regional volume to TIV, multiplied by a factor of 100. Statistical analysis was conducted using the chi-square tests for categorical variables and independent-sample t tests for continuous variables; no difference was obtained for any variables (*P* > 0.05). Data normality was tested using the Shapiro-Wilcoxon normality test, rejecting normality at *p* < 0.05.

Abbreviations: SD, standard deviation; APOE, apolipoprotein E; MMSE, mini-mental state examination; NFL, neurofilament light; pTau181, phosphorylated-tau181; MRI, magnetic resonance imaging; V, volume; L, left; R, right; T, thickness; MCI, mild cognitive impairment; NC, cognitively normal control; TIV, total intracranial volume; WMH, white matter hyperintensities.

**Table S2. Baseline Characteristics of the CSF Validation Cohorts**

|  | **Subset of Cohort 1**  **(n = 378)** | **Subset of Cohort 2**  **(n = 84)** | **Cohort 3**  **(n = 1303)** |
| --- | --- | --- | --- |
| **Demographics** |  |  |  |
| Age, mean (SD), y | 71.82 (7.391) | 74.74 (7.106) | 73.63 (7.330) |
| Men | 207 (54.8%) | 32 (38.1%) | 676 (51.9%) |
| Education, mean (SD), y | 16.27 (2.594) | 16.15 (2.529) | 16.33 (2.598) |
| ***APOE* ε4 allele** |  |  |  |
| ε4 -/- | 212 (56.1%) | 61 (72.6%) | 743 (57.0%) |
| ε4 +/- | 128 (33.9%) | 22 (26.2%) | 448 (34.4%) |
| ε4 +/+ | 38 (10.1%) | 1 (1.2%) | 112 (8.6%) |
| **MMSE, mean (SD), score** | 28.07 (1.758) | 29.06 (1.236) | 27.45 (3.043) |
| **Plasma biomarkers** |  |  |  |
| NFL, mean (SD), pg/mL | 38.26 (25.656) | 39.31 (18.260) | 40.41 (24.197) |
| pTau181, mean (SD), pg/mL | 16.92 (9.399) | 16.95 (9.499) | 18.34 (15.907) |
| **AD CSF core biomarkers** |  |  |  |
| Aβ, mean (SD), pg/mL | 1143.81 (597.878) | 1328.11 (566.478) | 1132.76 (616.919) |
| pTau, mean (SD), pg/mL | 25.70 (13.836) | 23.08 (9.762) | 26.98 (14.172) |
| tTau, mean (SD), pg/mL | 270.43 (123.855) | 253.54 (90.341) | 384.66 (130.185) |
| A+ | 192 (50.8%) | 30 (35.7%) | 679 (52.1%) |
| T+ | 129 (34.1%) | 24 (28.6%) | 504 (38.7%) |
| AD+ | 99 (26.2%) | 14 (16.7%) | 381 (29.2%) |
| **Structural MRI** |  |  |  |
| V_L_Hippocampus, mean (SD), %^§^ | 0.232 (0.0464) | 0.248 (0.0333) | 0.229 (0.0441) |
| V_R_Hippocampus, mean (SD), %^§^ | 0.238 (0.0440) | 0.247 (0.0335) | 0.233 (0.0444) |
| V_L_Amygdala, mean (SD), %^§^ | 0.089 (0.0190) | 0.091 (0.0144) | 0.087 (0.0182) |
| V_R_Amygdala, mean (SD), %^§^ | 0.094 (0.0192) | 0.095 (0.0152) | 0.091 (0.0182) |
| T_L_Inferior Temporal Cortex, mean (SD), mm | 2.707 (0.1992) | 2.708 (0.2213) | 2.672 (0.2252) |
| T_R_Inferior Temporal Cortex, mean (SD), mm | 2.759 (0.1946) | 2.746 (0.2045) | 2.722 (0.2276) |
| **Diagnosis at baseline** | MCI | NC | NC: 454 (34.8%)  MCI: 635 (48.7%)  Dementia: 211 (16.2%) |
| **Conversion after 36 months** | Dementia: 75 (19.8%) | MCI: 16 (19.0%)  Dementia: 8 (9.5%) | NA |

Mark §: volume of the hippocampus and amygdala was presented as the ratio of the regional volume to TIV, multiplied by a factor of 100. Participants were classified as having high brain Aβ loads (A+) or fibrillar tau (T+) according to a priori principles; AD+ means both A+ and T+.

Abbreviations: SD, standard deviation; APOE, apolipoprotein E; MMSE, mini-mental state examination; NFL, neurofilament light; pTau181, phosphorylated-tau181; AD, Alzheimer’s disease; CSF, cerebrospinal fluid; Aβ, amyloid-β; p-Tau, phosphorylated tau; tTau, total tau; MRI, magnetic resonance imaging; V, volume; L, left; R, right; T, thickness; MCI, mild cognitive impairment; NC, cognitively normal control; TIV, total intracranial volume; NA, not available.

**Table S3. Univariable analysis of predictors associated with dementia conversion for patients with MCI in the derivation cohort**

| **Candidate predictors** | **Median (IQR) or N (%)** | **OR (95% CI)** | ***P* value** |
| --- | --- | --- | --- |
| Age | 72.84 (67.59-78.27) | 1.042 (1.017-1.068) | 0.001 |
| Sex |  |  |  |
| Male | 432/761 | 1 (reference) |  |
| Female | 329/761 | 1.049 (0.722-1.520) | 0.799 |
| Education | 16.00 (14.00-18.00) | 0.912 (0.851-0.977) | 0.008 |
| *APOE* ε4 allele |  |  |  |
| ε4 -/- | 438 | 1 (reference) |  |
| ε4 +/- | 253 | 2.868 (1.907-4.342) | < 0.001 |
| ε4 +/+ | 70 | 4.239 (2.360-7.521) | < 0.001 |
| MMSE | 28.00 (27.00-30) | 0.662 (0.597-0.732) | < 0.001 |
| Plasma NFL levels | 34.10 (25.80-45.60) | 1.017 (1.009-1.026) | < 0.001 |
| Plasma pTau181 levels | 15.182 (10.376-21.652) | 1.049 (1.031-1.067) | < 0.001 |
| WMH | 3.858 (1.595-9.931) | 1.018 (0.995-1.040) | 0.107 |
| Infarcts (any locations) |  |  |  |
| no infarcts | 526/575 | 1 (reference) |  |
| infarcts | 49/575 | 0.897 (0.380-1.879) | 0.787 |
| Number of infarcts |  |  |  |
| no infarcts | 526/575 | 1 (reference) |  |
| single | 44/575 | 1.021 (0.429-2.162) | 0.959 |
| multiple | 5/575 | 0.000 (0.000-infinity) | 0.983 |
| Superficial infarcts |  |  |  |
| no infarcts | 526/575 | 1 (reference) |  |
| Superficial infarcts | 10/575 | 1.970 (0.419-7.227) | 0.332 |
| Infarcts of other locations | 39/575 | 0.676 (0.227-1.629) | 0.426 |
| Thalamic infarcts |  |  |  |
| no infarcts | 526/575 | 1 (reference) |  |
| Thalamic infarcts | 1/575 | 0.000 (0.000-infinity) | 0.982 |
| Infarcts of other locations | 48/575 | 0.919 (0.389-1.930) | 0.835 |
| Cortical infarcts |  |  |  |
| no infarcts | 526/575 | 1 (reference) |  |
| Cortical infarcts | 3/575 | 2.298 (0.106-24.228) | 0.499 |
| Infarcts of other locations | 46/575 | 0.825 (0.329-1.793) | 0.651 |
| Subcortical white matter infarcts |  |  |  |
| no infarcts | 526/575 | 1 (reference) |  |
| Subcortical white matter infarcts | 26/575 | 0.383 (0.061-1.320) | 0.197 |
| Infarcts of other locations | 23/575 | 1.622 (0.573-4.021) | 0.322 |
| Subcortical gray matter infarcts |  |  |  |
| no infarcts | 526/575 | 1 (reference) |  |
| Subcortical gray matter infarcts | 14/575 | 1.253 (0.280-4.106) | 0.733 |
| Infarcts of other locations | 35/575 | 0.766 (0.256-1.865) | 0.591 |
| Infratentorial infarcts |  |  |  |
| no infarcts | 526/575 | 1 (reference) |  |
| Infratentorial infarcts | 9/575 | 1.313 (0.194-5.535) | 0.737 |
| Infarcts of other locations | 40/575 | 0.811 (0.300-1.856) | 0.647 |
| V_L_Amygdala^§^ | 0.0878 (0.0776-0.1001) | 0.000 (0.000-0.000) | < 0.001 |
| V_R_Amygdala^§^ | 0.0945 (0.0821-0.1040) | 0.000 (0.000-0.000) | < 0.001 |
| V_L_Hippocampus^§^ | 0.2311 (0.2016-0.2617) | 0.000 (0.000-0.000) | < 0.001 |
| V_R_Hippocampus^§^ | 0.2357 (0.2090-0.2665) | 0.000 (0.000-0.000) | < 0.001 |
| T_L_Entorhinal Cortex | 3.381 (3.000-3.642) | 0.258 (0.175-0.376) | < 0.001 |
| T_R_Entorhinal Cortex | 3.493 (3.117-3.804) | 0.301 (0.214-0.419) | < 0.001 |
| T_L_Fusiform | 2.625 (2.504-2.736) | 0.030 (0.010-0.086) | < 0.001 |
| T_R_Fusiform | 2.633 (2.499-2.765) | 0.014 (0.005-0.040) | < 0.001 |
| T_L_Inferior Parietal Cortex | 2.322 (2.209-2.419) | 0.033 (0.010-0.104) | < 0.001 |
| T_R_Inferior Parietal Cortex | 2.356 (2.239-2.460) | 0.029 (0.009-0.090) | < 0.001 |
| T_L_Inferior Temporal Cortex | 2.722 (2.570-2.837) | 0.021 (0.007-0.057) | < 0.001 |
| T_R_Inferior Temporal Cortex | 2.761 (2.619-2.877) | 0.014 (0.005-0.040) | < 0.001 |
| T_L_Middle Temporal Cortex | 2.731 (2.610-2.852) | 0.032 (0.011-0.085) | < 0.001 |
| T_R_Middle Temporal Cortex | 2.776 (2.662-2.894) | 0.039 (0.013-0.106) | < 0.001 |
| T_L_Parahippocampal Gyrus | 2.691 (2.392-2.946) | 0.392 (0.241-0.635) | < 0.001 |
| T_R_Parahippocampal Gyrus | 2.645 (2.417-2.858) | 0.254 (0.142-0.450) | < 0.001 |

Data are n (%) or median (IQR). Notably, the statistical information of variables related to WMH and infarction was conducted in a subset of cohort 1 (n = 575). Mark §: volume of the hippocampus and amygdala was presented as the ratio of the regional volume to TIV, multiplied by a factor of 100.

Abbreviations: IQR, interquartile range; OR, odds ratio; CI, confidence interval; APOE, apolipoprotein E; MMSE, mini-mental state examination; NFL, neurofilament light; pTau181, phosphorylated-tau181; WMH, white matter hyperintensities; V, volume; L, left; R, right; T, thickness; TIV, total intracranial volume.

**Table S4. Predicted risk of dementia conversion according to the model score**

| **Points** | **N (%)** | **Estimated risk** |
| --- | --- | --- |
| 0.0 | 3.68% | 1.52% |
| 0.5 | 1.71% | 1.86% |
| 1.0 | 5.52% | 2.26% |
| 1.5 | 3.55% | 2.74% |
| 2.0 | 7.49% | 3.33% |
| 2.5 | 5.26% | 4.04% |
| 3.0 | 7.36% | 4.89% |
| 3.5 | 3.81% | 5.91% |
| 4.0 | 5.12% | 7.12% |
| 4.5 | 6.83% | 8.56% |
| 5.0 | 5.91% | 10.27% |
| 5.5 | 5.26% | 12.26% |
| 6.0 | 4.60% | 14.58% |
| 6.5 | 5.12% | 17.25% |
| 7.0 | 3.55% | 20.29% |
| 7.5 | 4.47% | 23.72% |
| 8.0 | 2.76% | 27.53% |
| 8.5 | 3.15% | 31.69% |
| 9.0 | 3.29% | 36.17% |
| 9.5 | 2.89% | 40.90% |
| 10.0 | 2.63% | 45.81% |
| 10.5 | 0.92% | 50.80% |
| 11.0 | 1.31% | 55.77% |
| 11.5 | 1.31% | 60.64% |
| 12.0 | 1.84% | 65.29% |
| 12.5 | 0.13% | 69.68% |
| 13.0 | 0.13% | 73.73% |
| 13.5 | 0.13% | 77.42% |
| 14.0 | 0.13% | 80.72% |
| 14.5 | 0.00% | 83.64% |
| 15.0 | 0.13% | 86.20% |
| 15.5 | 0.00% | 88.41% |
| 16.0 | 0.00% | 90.31% |
| 16.5 | 0.00% | 91.92% |
| 17.0 | 0.00% | 93.29% |

**Table S5. ROC curves**

| **Cohort** | **Dependent variable** | **AUC (95% CI, boot = 1000)** |
| --- | --- | --- |
| Cohort 3 (subset of NC) | A- vs. A+ | 0.668 (0.615 to 0.724) |
| Cohort 3 (subset of NC) | T- vs. T+ | 0.641 (0.585 to 0.701) |
| Cohort 3 (subset of NC) | AD- vs. AD+ | 0.692 (0.611 to 0.771) |
| Cohort 3 (subset of MCI) | A- vs. A+ | 0.784 (0.751 to 0.816) |
| Cohort 3 (subset of MCI) | T- vs. T+ | 0.765 (0.727 to 0.802) |
| Cohort 3 (subset of MCI) | AD- vs. AD+ | 0.808 (0.770 to 0.843) |
| Cohort 3 (subset of Dementia) | A- vs. A+ | 0.727 (0.607 to 0.846) |
| Cohort 3 (subset of Dementia) | T- vs. T+ | 0.662 (0.579 to 0.743) |
| Cohort 3 (subset of Dementia) | AD- vs. AD+ | 0.665 (0.588 to 0.741) |

Participants were classified as having high brain Aβ loads (A+) or fibrillar tau (T+) according to a priori principles; AD+ means both A+ and T+.

Abbreviations: ROC, receiver operating characteristic; AUC, area under the curve; CI, confidence interval; AD, Alzheimer’s disease; MCI, mild cognitive impairment; NC, cognitively normal control.
